# Molecular detection of *Babesia* and *Theileria* from crossbred cattle in Sirajganj and Rangpur districts of Bangladesh

**DOI:** 10.1002/vms3.989

**Published:** 2022-11-04

**Authors:** Md. Jakir Hossain, Sanjana Raut, Rahul Pratap Singh, Pravin Mishra, Md. Shahadat Hossain, Anita Rani Dey, Ajran Kabir, Anisuzzaman, Md. Hasanuzzaman Talukder, Md. Shahiduzzaman

**Affiliations:** ^1^ Department of Parasitology Bangladesh Agricultural University Mymensingh Bangladesh; ^2^ Department of Microbiology & Hygiene Bangladesh Agricultural University Mymensingh Bangladesh

**Keywords:** bovine piroplasmosis, cattle, *Babesia*, *Theileria*, PCR, nested PCR

## Abstract

**Background:**

*Babesia* and *Theileria* are potential threats to the livestock industry, causing considerable economic losses. These tick‐borne blood parasites are more prevalent in crossbred cattle than local cattle in Bangladesh.

**Objectives:**

To confirm the species of *Babesia* and *Theileria* in crossbred cattle from the northern part of Bangladesh using conventional and molecular tools.

**Methods:**

A total of 385 crossbred cattle blood samples were subjected to DNA extraction and PCR. For molecular detection, *B. bigemina* rhoptry‐associated protein 1a, *B. bovis* spherical body protein‐4, and *Theileria* spp. 18S rRNA were used as the marker genes.

**Results:**

Using PCR, only 72 (18.7%) samples were found piroplasm positive, of which 12.2% *Theileria*, 4.7% *Babesia*, and 1.8% mixed infections. Both *Babesia* (7.3%), *Theileria* (7.7%) and mixed (2.8%) infections were detected in Sirajganj, and only *Theileria* (20.4%) was detected in Rangpur district. By PCR and nPCR we detected *B. bigemina* and *T. annulata* in Sirajganj district, and *Theileria* sp. in Rangpur district. The target gene sequences of isolated pathogens confirmed *B. bigemina* and *T. annulata*, and *Theileria* sp from these samples. Blood smears of all samples were also examined microscopically for *Babesia* and/or *Theileria* spp. and 14.3% of samples were found positive, of which 5.9% *Babesia* and 8.3% *Theileria*. Generally, the pathogens detected in Sirajgang and Rangpur were genetically related to South Asia, particularly South East Asian isolates.

**Conclusions:**

These findings provide information for a better understanding of the epidemiology of *Babesia* and *Theileria* as well as to improve the approaches for diagnosis and control of tick‐borne diseases in Bangladesh.

## Graphical abstract

By using molecular tools 12.2% *Theileria*, 4.7% *Babesia*, and 1.8% mixed infections were detected.

*B. Bigemina* isolates (MH790974 Sirajganj) *T. annulata* (LC439356 Sirajganj) are highly conserved species and *Theileria* sp (LC419995.1 Rangpur) was found as a different genetic clade which may introduced from neighboring India and Myanmar

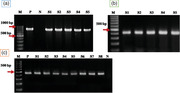

## 1 INTRODUCTION

Babesiosis and theileriosis cause significant economic losses in the tropics and subtropics (Inci et al., 2007; Uilenberg, 1995). The economic losses are due to loss of body weight, milk output and animal death, as well as indirectly for the expenses related to therapeutic management of clinical cases and targeting control campaigns (Guswanto et al., 2017). In Bangladesh, babesiosis (2.3–9.0%) and theileriosis (5.8–8.5%) are found in both local and crossbred cattle (Al Mahmud et al., 2015; Alim et al., 2012; Hosen et al., 2020; Roy et al., 2018; Samad et al., 1983). Crossbred cattle had a greater prevalence of bovine piroplasmosis than local cattle (Siddiki et al., 2010). These tick‐borne blood parasites are prevalent in crossbred cattle rearing in Sirajganj and Rangpur districts (Islam et al., 2006). Among the cattle ticks, *Rhipicephalus* (*Boophilus) microplus, Haemaphysalis bispinosa*, *Hyalomma truncatum, H. anatolicum Amblyomma gervaisi, H. canestrini, Haemaphysalis kinneari*, *R. sanguineus*, *Rhipicephalus evertsi evertsi* and *Amblyomma variegatum*, are prevalent in Bangladesh (Ghosh et al., 2007; Kabir et al., 2011; Shuvo et al., 2021). However, the geo‐climatic conditions of Bangladesh are thought to be very conducive to a wide variety of parasites, including ticks (Dey et al., 2020). The presence of a variety of tick species enhances the transmission of tick‐borne diseases, especially babesiosis and theileriosis.

The diagnosis of these important blood protozoa are mainly based on clinical symptoms and microscopic examination of Giemsa‐stained blood smears in Bangladesh. Several researchers identified bovine piroplasms using microscopic examination for epidemiological investigation (Al Mahmud et al., 2015; Chowdhury, Hossain et al., 2006). Limited studies also have performed serological examination to confirm the tentative diagnosis of infections (Ali et al., 2016; Rahman, Sumon et al., 2015). However, these studies are only reliable for the diagnosis of acute cases but have limited value in subclinical cases due to low parasitaemia (Alim et al., 2012; Hosen et al., 2020; Samad et al., 1983). Compared to serological testing and microscopic identification of piroplasms, molecular detection of piroplasms infection by polymerase chain reaction (PCR) has proven to be very sensitive, accurate, and the gold standard test particularly in detecting *Babesia* and *Theileria* in carrier cattle (Belotindos et al., 2014).

*B. bigemina* rhoptry‐associated protein 1a (RAP‐1a) is a highly conserved gene among *B. bigemina* isolates that has been utilised in several studies (Guswanto et al., 2017; Ibrahim et al., 2013; Moumouni et al., 2015; Terkawi et al., 2012). The *B. bovis* spherical body protein‐4 (SBP‐4) gene is conserved between geographical isolates and has little homology with other apicomplexan parasites, emphasising the gene's usefulness as a particular target for molecular diagnosis (de Vries et al., 2006; Ruef et al., 2000). On the other hand, the 18S rRNA gene has been widely used for the identification and phylogenies of *Theileria* (Bawm et al., 2021; Mohamed et al., 2018).

In recent years, molecular studies have been performed to detect piroplasmosis in cattle of Mymensingh (Roy et al., 2018) and Rajshahi district (Le Huy et al., 2020) of Bangladesh. However, these studies were on a few tick‐borne diseases with inadequate information on their genotypes or even knowledge on their molecular epidemiology, which is critical for the control and prevention of these diseases.

Molecular identification, with or without clinical indications of bovine piroplasmosis, provides an alternative method for the direct detection of piroplasms in carrier animals to overcome economic losses. Therefore, in the present study, a molecular survey of *Babesia* and *Theileria* species based on PCR amplification was conducted in the cattle population of the Sirajganj and Rangpur districts of Bangladesh.

## 2 MATERIALS AND METHODS

### 2.1 Study area

**FIGURE 1:**
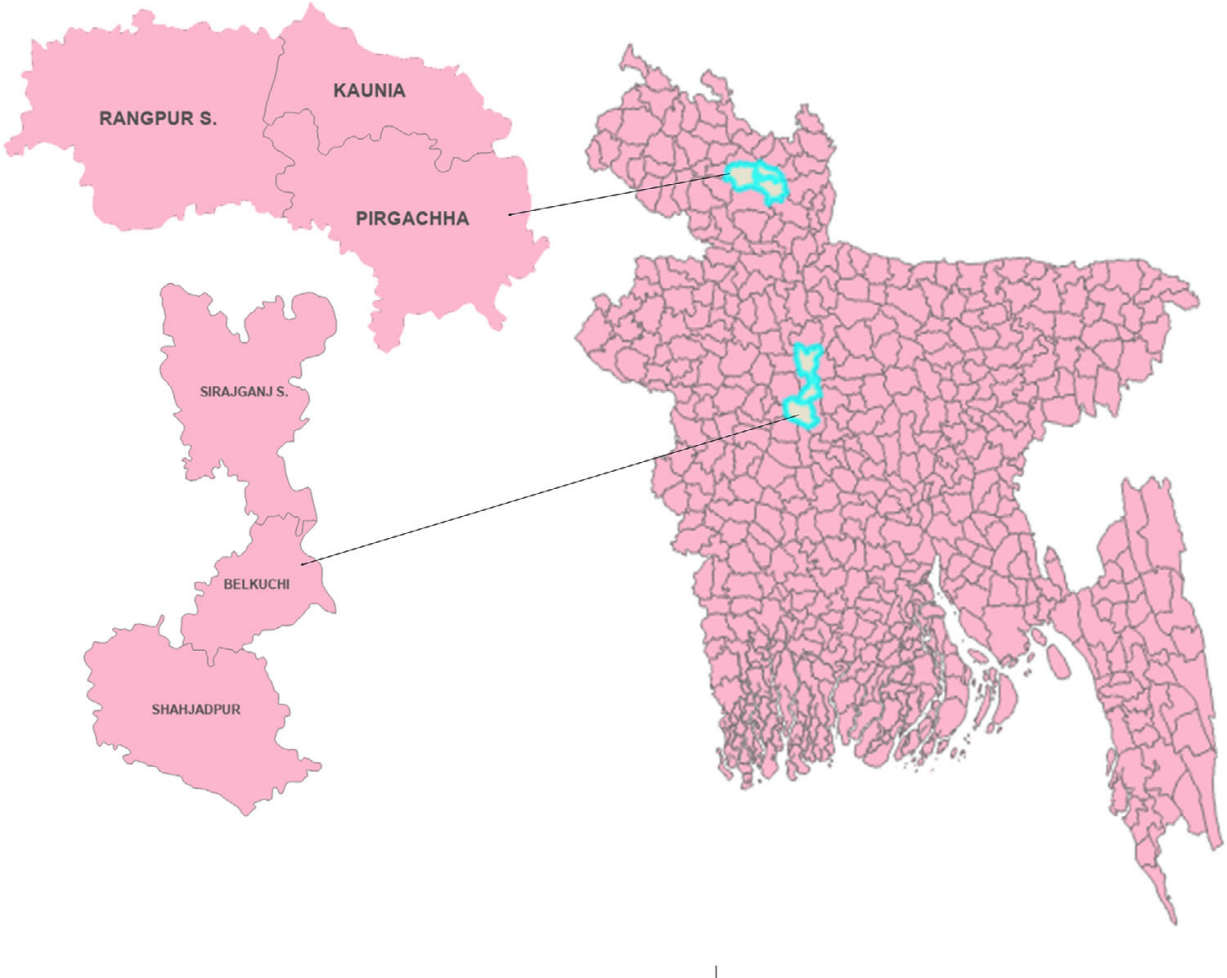
Map of Bangladesh indicating different upazilas of Sirajganj and Rangpur districts from where the samples were collected

This study was conducted in two northern districts (Sirajganj and Rangpur) of Bangladesh. Sirajganj is located in the north‐central region (24° 27'0"N, 89° 45'0"E) of Bangladesh, besides the Jamuna river. The selected areas in the Sirajganj district were Belkuchi, Shahjadpur, and Sirajganj Sadar, the largest bathan (a large area of grazing where cattle are housed and maintained) for ruminants is present in Shahjadpur upazila (a subunit of a district). ‘Baghabari Milk Vita’, the largest public‐private milk producing company, is located in the same area. Rangpur, on the other hand, is located in the northwestern region of Bangladesh (25° 33′ 36′′ N, 89° 15′ 0′′ E), on the bank of the Ghaghat river. The selected areas were Kaunia, Pirgacha and Rangpur Sadar upazilas (Figure 1).

### 2.2 Sample collection

Blood samples were collected from randomly selected 385 crossbred (local breed crossed with Holstein‐Friesian and Sahiwal) cattle from 80 different dairy farms. Of these samples, 248 were from Sirajganj and 137 from Rangpur district, Bangladesh. The cattle were reared in semi‐intensive and free‐range systems (bathan). Blood samples were collected by ear puncture in 4 ml EDTA‐coated tubes. A few drops of blood were immediately used to prepare thin blood smears, air dried, fixed in methyl alcohol for 2 min and brought to the laboratory for further processing. The remaining samples were kept at –20°C until DNA extraction.

### 2.3 Microscopic examination

Thin blood smears were prepared, fixed with methanol, and stained with 10% Giemsa stain for 30 min. After staining, slides were washed with tap water and examined at 100× objective magnification by adding immersion oil. Each sample was examined in triplicate.

### 2.4 DNA extraction

Genomic DNA was extracted and purified from 200 μl of the blood of both positive and negative samples using the Purelink TM^®^ DNA blood Mini kit (Invitrogen, USA) according to the manufacturer's instructions. The concentration of the extracted DNA was measured using a nanodrop (NanoDrop™, Thermo Fisher, USA).

### 2.5 Molecular detection of *Babesia* and *Theileria* spp

To confirm the *Babesia*, PCR was conducted targeting the amplification of *rhoptry‐associated protein‐1a* (*RAP‐1a*) gene of *B. bigemina* and *spherical body protein‐4* (*SBP*‐4) of *B. bovis* through PCR and nPCR using species‐specific primers (Table 1). To detect *B. bigemina*, PCR was performed with the following cyclic conditions: 95°C for 5 min, 35 cycles at 94°C for 1 min, 55°C for 1 min, 72°C for 1 min, and a final extension of 72°C for 10 min (Terkawi et al., 2011). Similar conditions were used in nPCR to amplify the 412 bp region of RAP‐1a gene of *B. bigemina* . For the detection of *B. bovis*, PCR conditions were as follows: initial denaturation at 94°C for 1 min, followed by 35 cycles of denaturation at 94°C for 30 s, annealing at 65°C for 1 min, extension at 72°C for 1 min, and a final extension at 72°C for 7 min. The conditions for nPCR were similar except for the annealing temperature, which was 53.5°C (Terkawi et al., 2011).

**TABLE 1:** Nucleotide sequences of the primers used for PCR analysis and the PCR product profile

**Pathogen target gene**	**Assay**	**Oligonucleotide sequences (5' > 3')**	**Product size (bp)**	**References**
*Babesia/Theileria*	PCR	F: 5′‐CCAATCCTGACACAGGGAGGTAGTGACA R: 5′‐CCCCAGAACCCAAAGACTTTGATTTCTCTCAAG	619	Kledmanee et al. (2009)
*B. bigemina RAP‐1a*	PCR	F: 5′‐GAGTCTGCCAAATCCTTAC R: 5′‐TCCTCTACAGCTGCTTCG	879	Terkawi et al. (2011)
	nPCR	F: 5′‐AGCTTGCTTTCACAACTCGCC R: 5′‐TTGGTGCTTTGACCGACGACAT	412	
*B. bovis* SBP‐4	PCR	F: 5′‐AGTTGTTGGAGGAGGCTAAT R: 5′‐TCCTTCTCGGCGTCCTTTTC	907	Terkawi et al. (2011)
	nPCR	F: 5′‐GAAATCCCTGTTCCAGAG R: 5′‐TCGTTGATAACACTGCAA	503	
*Theileria* 18S rRNA	PCR	F: 5′‐CGGTAATTCCAGCTCCAATAGCGT‐3′ R: 5′‐TTTCTCTCAAAGGTGCTGAAGGAGT‐3′	480	Wamuyu et al. (2015)

*Theileria* species were identified through the amplification of the *18S rRNA* gene. The reaction conditions were 95°C for 5 min, 30 cycles at 95°C for 30 s, 54°C for 30 s, 72°C for 1 min, and a final extension of 72°C for 9 min (Wamuyu et al., 2015).

Double distilled water was used as a negative control for all PCR. The PCR was done in an Applied Biosystems^TM^ 2720 thermal cycler. The amplified product of PCR assay was analysed by gel electrophoresis on a 1.5% agarose gel and stained with ethidium bromide for visualisation of gel under UV illumination.

### 2.6 Sequencing of DNA

The species of *Babesia* and *Theileria* were confirmed by sequencing the representative PCR products obtained from nPCR. DNA fragments of approximately 412 bp of *Babesia bigemina RAP‐1a* gene and 480 bp of *Theileria 18S rRNA* gene were purified from 1.5% agarose gel using a commercial kit (Wizard SV gel and PCR clean‐up system, Promega, Madison, WI, USA). The sequencing was performed at DNA Solution Ltd, Dhaka, Bangladesh, by using Bigdye terminator sequencing kit in ABI 3500 Dx Genetic analyser (Applied Biosystems, USA).

### 2.7 Phylogenetic analysis

The sequences of *ITS‐1* were aligned using the program Clustal W within MEGA v.6.0 (Tamura et al., 2011). The sequence results were compared with the available sequence in the GenBank database using the Basic Local Alignment Search Tool (BLAST) (https://blast.ncbi.nlm.nih.gov/Blast.cgi). The phylogenetic trees were constructed using the neighbour‐joining method in MEGA version 6.0 (Tamura et al., 2013). Bootstrap analysis with 1000 replications was used to estimate the confidence levels of the branching patterns of the trees. Representative sequences that were identified in this study were deposited in the GenBank. The gene sequences from GenBank can be retrieved with accession number for *Babesia* (MH790974.1 *Babesia bigemina* Sirajganj) and *Theileria* (LC419995.1‐*Theileria* sp. Rangpur; LC439356.1‐*Theileria annulata*, Sirajganj).

## 3 RESULTS

### 3.1 Microscopic detection of *Babesia* and *Theileria*

**FIGURE 2:**
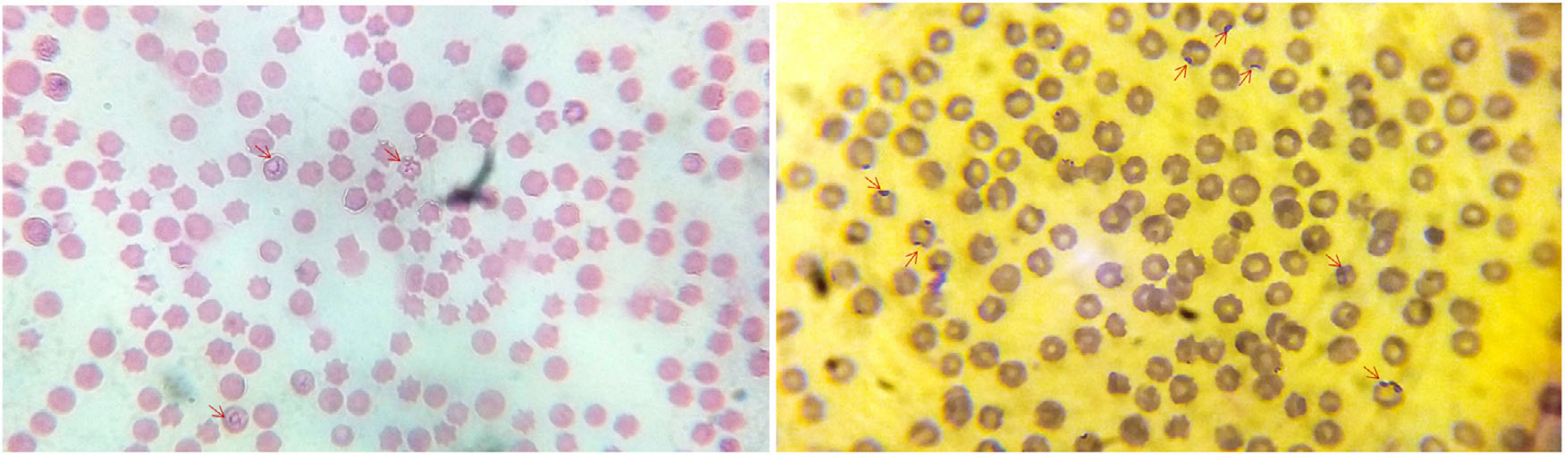
Arrow indicating *Babesia* spp. (left) and *Theileria* spp. (right) within RBC in Giemsa stained blood smears made from cattle (100×)

A total of 385 blood samples (248 samples from Sirajganj and 137 samples from Rangpur district) were collected and microscopically examined to detect *Babesia* or *Theileria* (Figure 2). Among the samples examined, a total of 55 (14.3%) were positive, of which 23 (5.9%) were *Babesia* and 32 (8.3%) were *Theileria*. Microscopic examination of thin blood smears from Sirajganj district revealed 9.3% (23/248) infection with *Babesia* and 5.2% (13/248) with *Theileria* (Table 2). Whereas 13.7% (19/137) infection with *Theileria* and no infection with *Babesia* were detected in samples from the Rangpur district (Table 2). Since it is hard to differentiate the species of *Babesia* and *Theileria* by microscopic examination, we confirmed the cases of either *Babesia* or *Theileria* or mixed infection by molecular tools.

**TABLE 2:** Prevalence of haemoprotozoa based on microscopy and PCR

		**Microscopic examination**			**PCR examination**			
**Name of districts**	**Name of upazilas (*n*)**	**Positive (%)**	***Babesia* (%)**	***Theileria* (%)**	**Positive (%)**	***B. bigemina* (%)**	***Theileria* spp. (%)**	**Mixed (%)**
Sirajganj	Belkuchi (83)	13 (15.6)	10 (12.0)	3 (3.6)	11 (13.2)	4 (4.8)	6 (7.2)	1 (1.2)
	Shahjadpur (100)	8 (8.0)	6 (8.0)	2 (2.0)	26 (26.0)	11 (4.8)	9 (9.0)	6 (6.0)
	Sirajganj Sadar (65)	15 (23.1)	7 (10.8)	8 (12.3)	7 (10.8)	3 (4.6)	4 (6.2)	0 (0.0)
	**Subtotal (*n* = 248)**	**36 (14.5)**	**23 (9.3)**	**13 (5.2)**	**44 (17.7)**	**18 (7.3)**	**19 (7.7)**	**7 (2.8)**
Rangpur	Kaunia (44)	7 (15.9)	0 (0.0)	7 (15.9)	12 (27.2)	0 (0.0)	12 (27.2)	0 (0.0)
	Pirgacha (61)	10 (16.3)	0 (0.0)	10 (16.3)	11 (18.0)	0 (0.0)	11 (18.0)	0 (0.0)
	Rangpur Sadar (32)	2 (6.3)	0 (0.0)	2 (6.3)	5 (15.6)	0 (0.0)	5 (15.6)	0 (0.0)
	**Subtotal (*n* = 137)**	**19 (13.9)**	**0 (0.0)**	**19 (13.9)**	**28 (20.4)**	**0 (0.0)**	**28 (20.4)**	**0 (0.0)**
	**Total (385)**	**55 (14.3)**	**23 (5.9)**	**32 (8.3)**	**72 (18.7)**	**18 (4.7)**	**47 (12.2)**	**7 (1.8)**

### 3.2 Molecular detection of *Babesia* and *Theileria* spp

**FIGURE 3:**
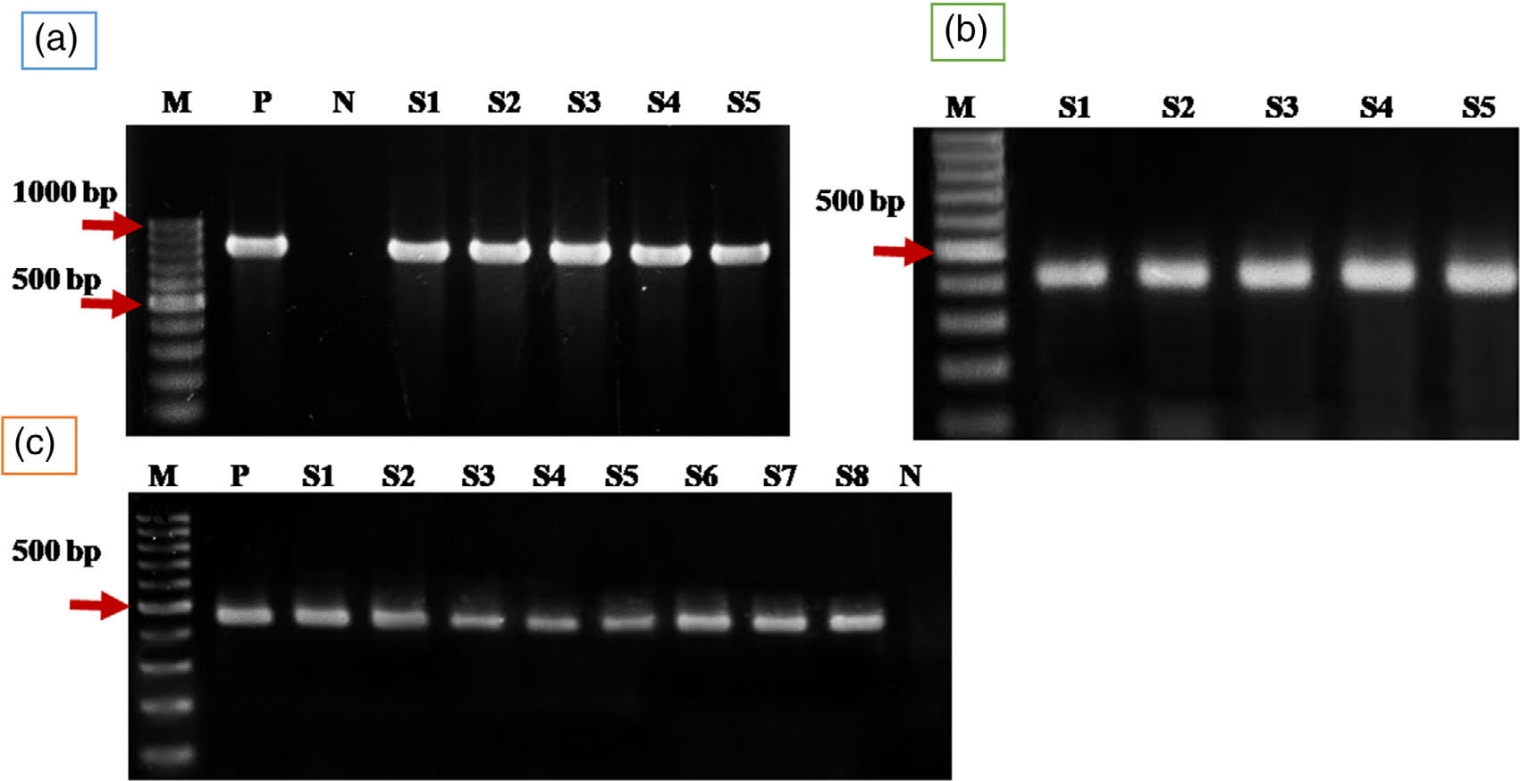
(a) Gel electrophoresis showing Primary PCR amplicons of the *Babesia bigemina RAP‐1a* gene fragment (879 bp). (b) nPCRamplicons from A (412 bp). (c) Gel electrophoresis showing PCR amplicons of the *Theileria* spp. *18S rRNA* gene fragment (480 bp). In lanes: M, molecular weight marker = 1 kbp; P, positive control; N, negative control; S1–S8 are samples. The sequence confirmed sample was used as positive control

Amplification of *B. bigemina RAP‐1a* gene fragment from all samples by primary PCR followed by nPCR provided 4.7% (18/385) positive cases for *B. bigemina* infection with an expected band size of 879 bp in primary PCR (Figure 3a) and 412 bp in nPCR (Figure 3b). Amplification of 18S rRNA gene for *Theileria* confirmed the presence of *Theileria* spp. (band size 480 bp) in 12.2% (47/385) samples (Figure 3c). Mixed infections of *Theileria* and *Babesia* were detected in 1.8% (7/385) samples by PCR which is found only in the Sirajganj district. The distribution of each species detected by PCR is presented in Table 2.

### 3.3 Blast analysis and sequence alignment

The NCBI‐BLAST analysis of *RAP‐1a* gene sequence of *B. bigemina* field isolates in the present study revealed 100% homology to the published *B. bigemina* isolates from Tanzania (MG210824), Uganda (MG426201), Kenya (KP347559), Turkey (KT220512), Egypt (KF192811) and the United States (AF017296).

The *18S rRNA* gene sequence of *T. annulata* identified in this study (LC439356‐*Theileria annulata*, Sirajganj) shared 100% identity and was homologous to the sequences of isolates from India (MK713333; MF287950), Myanmar (LC576818), Malaysian (KJ917961), Indonesia (AB000274) and China (MN252454).

The nucleotide sequence identity value of *18S rRNA* gene sequences of *Theileria* (LC419995‐*Theileria* sp.) isolated from the Rangpur district was only 91.81%, similar to the sequence of *T. annulata* from India (LC419995; MF287950), *Theileria* sp. C58T of Malaysia (KJ917960), *T. orientalis* of Myanmar (LC576818), *Theileria* sp. of Indonesia (AB000274) and China (DQ286801).

### 3.4 Phylogenetic analysis

**FIGURE 4:**
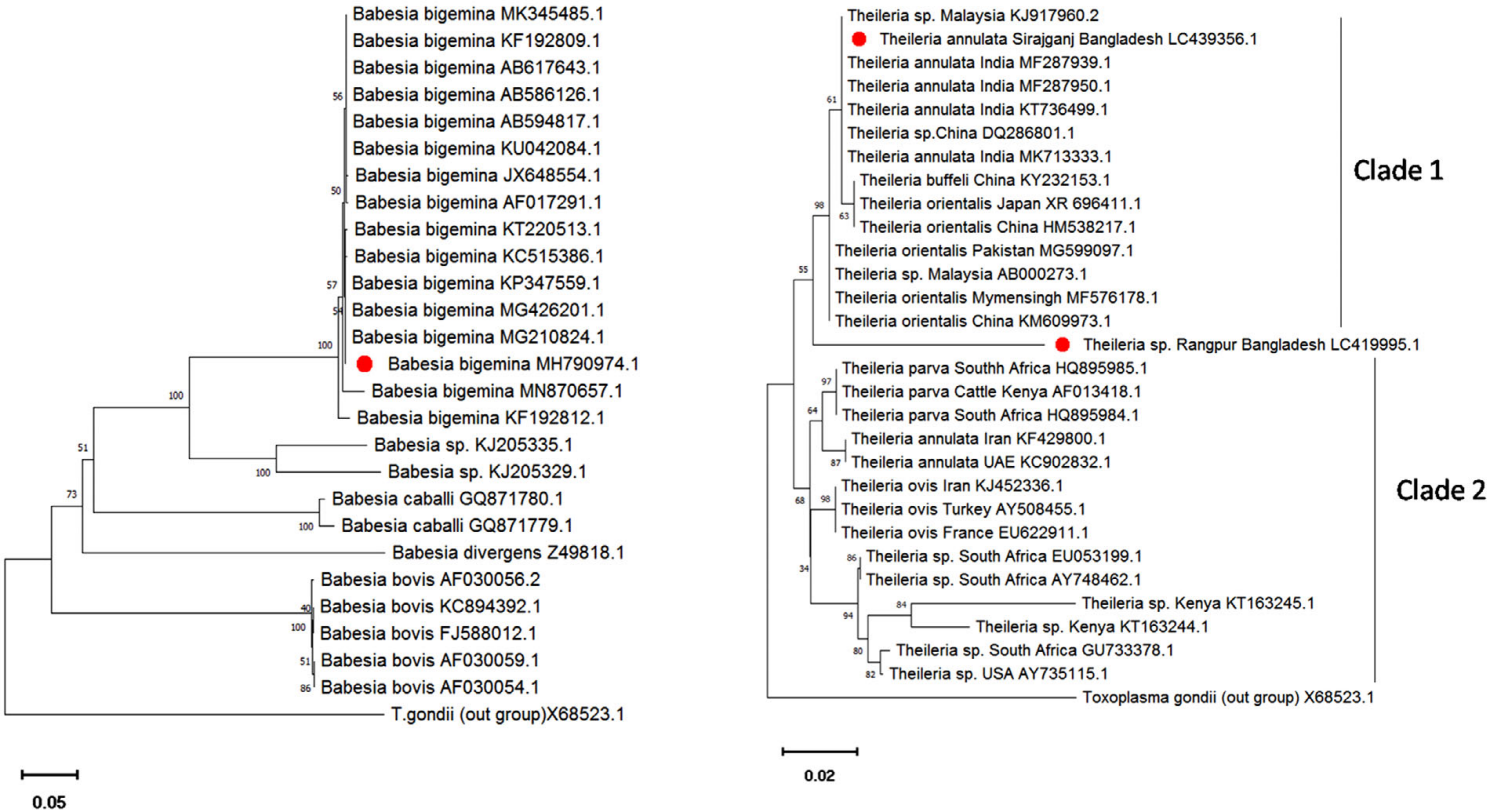
Neighbour‐joining analysis of the RAP‐1a gene sequences showing the phylogenetic relationship of *Babesia* (Left) and 18s rRNA gene of *Theileria* (Right) with reference sequences. *Toxoplasma gondii* was used as out‐group. Red bullet indicates studied sequences. Branch lengths are proportional to the estimated genetic distance between sequences and numbers above the branches indicate bootstrap values based on 1000 replicates

The *RAP‐1a* gene sequences of *B. bigemina*, *Babesia* sp., *B. caballi*, *B. divergens* and *B. bovis* retrieved from the GenBank were used in phylogenetic analyses. The neighbour‐joining phylogenetic analysis of 27 sequences disclosed that *B. bigemina* isolates (MH790974.1) identified in the present study clustered together with previously described *B. bigemina* isolates with strong nodal support where *Toxoplasma gondii* was used as out‐group. Again, a comparison of branch lengths between *B. bigemina* (MH790974 Sirajgangj) and accepted species shows that *B. bigemina* (MH790974 Sirajganj) likely represents a valid species (Figure 4).

Phylogenetic tree was structured with *18S rRNA* gene sequences of *Theileria* isolates from different countries. Two distinct clades were observed where clade 1 represents the sequences of *Theileria* from Asian countries and clade 2 represents the sequences of *Theileria* from African countries. *T. annulata* (LC439356 Sirajganj) formed a cluster in Asian clade with the previous sequences of *T. annulata* with 61% nodal support. Conversely, *Theileria* sp. (LC419995.1 Rangpur) was far from the Asian clade but not in the African clade (Figure 4).

## 4 DISCUSSION

Bovine babesiosis and theileriosis are responsible for more than 50% of losses in crossbred cattle farming (Chaudhry et al., 2010). *Babesia* and *Theileria* infections in carrier cattle or subclinical hosts are difficult to detect because of the very low parasitaemia. But the diagnosis of carrier animals in farms or herds is essential for preventing tick‐borne transmission to healthy animals.

The selected study areas are densely populated with cattle. Sirajganj is recognised as the most important dairy cattle rearing area, with the highest number of dairy farms according to the district statistics of Sirajganj, Bangladesh. On the other hand, Rangpur is also popularly known for smallholder cattle farms owned by marginal farmers. Most of the studies on bovine piroplasmosis conducted previously had been performed in these two districts, which reported the presence of these tick‐borne parasitic diseases in these areas (Aliet al., 2016; Rahman et al., 2015). However, these studies were conducted to detect bovine piroplasmosis based on microscopy and PCR without any confirmation of the species.

The present study investigated the samples from crossbred cattle rearing in the selected farms or *bathan*, which provided clear data for understanding the epidemiology of tick‐borne diseases in Bangladesh. The detection rates of *Babesia* and *Theileria* were higher in PCR methods (18.7%) compared to microscopy (14.3%), indicating the significance of using molecular tools to detect carrier hosts for effective control strategies in Bangladesh. The overall positive cases were higher in Rangpur (20.4%) compared to Sirajganj (17.7%) (Table 2). Chowdhury et al. (2006) recorded that the prevalence of *B. bigemina* infection in cattle was 3.30% in the Sirajganj Sadar area of Bangladesh. Al Mahmud et al. (2015) recorded that the overall prevalence of babesiosis and theileriosis of cattle in the Sirajganj district was 2.27% and 5.82%, respectively. These lower prevalence rates compared to this study (7.3% *Babesia*, 7.7% *Theileria*) might be due to microscopic examination of the samples. On the other hand, the prevalence of *Theileria* was higher (20.4%) in Rangpur compared to Sirajganj (7.7%), which is in accordance with the report of Samad et al. (1983), who observed that the overall 22.03% prevalence of theileriosis in dairy cattle of Bangladesh.

The area of Sirajganj from where blood samples were collected is known as *bathan* (low‐lying large grazing areas) with thousands of farms (small to medium) where mainly crossbreds are reared for milking purposes. During the time of spring or immediately after the monsoon, farmers graze their cows in *bathan*. Anti‐Babesial or anti‐Theilerial drugs are commonly used to treat the symptomatic animals but due to the availability of hard ticks (*Rhipicephalus/ Boophilus*) and asymptomatic carrier animals, the infections are more common in this area. Abundant access to grazing land may increase the probability of cattle coming into contact with ticks and thereafter may increase the chance of infection with *Babesia* (Roy et al., 2018).

The phylogenetic analysis of *RAP‐1* gene sequence represents *B. Bigemina* isolates (MH790974) identified in the present study clustered together with previously described *B. bigemina* isolates, indicating the species is highly conserved.

In this study, *T. annulata* formed a cluster in Asian clade with the previously identified *T. annulata* whereas *Theileria* sp. identified from Rangpur formed a different genetic clade from clade 1 (Asian) and clade 2 (African). However, *Theileria* sp. identified in this study is closely related to *T. annulata 18S ribosomal RNA* gene isolates from India and Myanmar. This finding suggests that a different genetic clade is introduced from neighbouring India and Myanmar because cattle are frequently imported from these two countries. Further studies are required to elucidate the origin, distribution, vector, and pathogenesis of *Theileria* parasites circulating in cattle in Bangladesh.

From our knowledge, no previous information was available on the polymorphism of *B. bigemina RAP‐1* sequences of Bangladesh, suggesting the need to collect more geographical *B. bigemina* isolates of Bangladesh for sequencing in order to test the level of diversity among *RAP‐1* sequences of *B. bigemina* isolates.

Since babesiosis and theileriosis are associated with the availability of ticks, therefore, routine diagnostic procedures, isolation of carrier animals from herds, and best management practices with clean environment are necessary for controlling these diseases in Bangladesh.

## 5 CONCLUSION

In the present study, 14.3% crossbred cattle were infected with *Babesia* (5.9%) and *Theileria* (8.3%) using microscopic examination. However, by employing molecular tools, 18.7% animals were found to be infected with *Babesia* (4.7%) and *Theileria* (12.2%) signifying the importance of molecular tools for proper investigating the bovine piroplasmosis in Bangladesh. The result also detected *B. bigemina*, *T. annulata* and *Theileria* sp. by amplifying target genes. It is recommended that more isolates from other geographical regions must be examined to provide more information for the genotyping of Bangladeshi isolates of *Babesia* and *Theileria*.

## AUTHOR CONTRIBUTIONS

Md. Jakir Hossain: investigation; methodology; writing – original draft. Sanjana Raut: investigation; methodology; writing – original draft. Rahul Pratap Singh: data curation; methodology; validation. Pravin Mishra: data curation; formal analysis; validation. Anita Rani Dey: formal analysis; resources; supervision. Ajran Kabir: formal analysis; investigation; methodology; validation. Anisuzzaman: supervision; visualisation; writing – review & editing.

## CONFLICT OF INTEREST

The authors declare that there is no conflict of interest.

### ETHICS STATEMENT

The authors tried to maintain the highest possible ethical standards in their works without any injury of animals. The study was approved by the Animal Welfare and Experimentation Ethical Committee of Bangladesh Agricultural University. The approval number is AWEEC/BAU/2017 (16).

### PEER REVIEW

The peer review history for this article is available at https://publons.com/publon/10.1002/vms3.989

## Data Availability

The data that support the findings of this study are available from the corresponding author upon reasonable request.
